# Developing a Long COVID Phenotype for Postacute COVID-19 in a National Primary Care Sentinel Cohort: Observational Retrospective Database Analysis

**DOI:** 10.2196/36989

**Published:** 2022-08-11

**Authors:** Nikhil Mayor, Bernardo Meza-Torres, Cecilia Okusi, Gayathri Delanerolle, Martin Chapman, Wenjuan Wang, Sneha Anand, Michael Feher, Jack Macartney, Rachel Byford, Mark Joy, Piers Gatenby, Vasa Curcin, Trisha Greenhalgh, Brendan Delaney, Simon de Lusignan

**Affiliations:** 1 Royal Surrey NHS Foundation Trust Guildford United Kingdom; 2 Nuffield Department of Primary Care Health Sciences University of Oxford Oxford United Kingdom; 3 Department of Clinical and Experimental Medicine University of Surrey Guildford United Kingdom; 4 Department of Surgery & Cancer Institute of Global Health Innovation Imperial College London London United Kingdom; 5 Population Health Sciences Kings College London London United Kingdom; 6 Royal College of General Practitioners Research and Surveillance Centre London United Kingdom

**Keywords:** medical record systems, computerized, Systematized Nomenclature of Medicine, postacute COVID-19 syndrome, phenotype, COVID-19, long COVID, ethnicity, social class, general practitioners, data accuracy, data extracts, biomedical ontologies, SARS-CoV-2, hospitalization, epidemiology, surveillance, public health, BioPortal, electronic health record, disease management, digital tool

## Abstract

**Background:**

Following COVID-19, up to 40% of people have ongoing health problems, referred to as postacute COVID-19 or long COVID (LC). LC varies from a single persisting symptom to a complex multisystem disease. Research has flagged that this condition is underrecorded in primary care records, and seeks to better define its clinical characteristics and management. Phenotypes provide a standard method for case definition and identification from routine data and are usually machine-processable. An LC phenotype can underpin research into this condition.

**Objective:**

This study aims to develop a phenotype for LC to inform the epidemiology and future research into this condition. We compared clinical symptoms in people with LC before and after their index infection, recorded from March 1, 2020, to April 1, 2021. We also compared people recorded as having acute infection with those with LC who were hospitalized and those who were not.

**Methods:**

We used data from the Primary Care Sentinel Cohort (PCSC) of the Oxford Royal College of General Practitioners (RCGP) Research and Surveillance Centre (RSC) database. This network was recruited to be nationally representative of the English population. We developed an LC phenotype using our established 3-step ontological method: (1) ontological step (defining the reasoning process underpinning the phenotype, (2) coding step (exploring what clinical terms are available, and (3) logical extract model (testing performance). We created a version of this phenotype using Protégé in the ontology web language for BioPortal and using PhenoFlow. Next, we used the phenotype to compare people with LC (1) with regard to their symptoms in the year prior to acquiring COVID-19 and (2) with people with acute COVID-19. We also compared hospitalized people with LC with those not hospitalized. We compared sociodemographic details, comorbidities, and Office of National Statistics–defined LC symptoms between groups. We used descriptive statistics and logistic regression.

**Results:**

The long-COVID phenotype differentiated people hospitalized with LC from people who were not and where no index infection was identified. The PCSC (N=7.4 million) includes 428,479 patients with acute COVID-19 diagnosis confirmed by a laboratory test and 10,772 patients with clinically diagnosed COVID-19. A total of 7471 (1.74%, 95% CI 1.70-1.78) people were coded as having LC, 1009 (13.5%, 95% CI 12.7-14.3) had a hospital admission related to acute COVID-19, and 6462 (86.5%, 95% CI 85.7-87.3) were not hospitalized, of whom 2728 (42.2%) had no COVID-19 index date recorded. In addition, 1009 (13.5%, 95% CI 12.73-14.28) people with LC were hospitalized compared to 17,993 (4.5%, 95% CI 4.48-4.61; *P*<.001) with uncomplicated COVID-19.

**Conclusions:**

Our LC phenotype enables the identification of individuals with the condition in routine data sets, facilitating their comparison with unaffected people through retrospective research. This phenotype and study protocol to explore its face validity contributes to a better understanding of LC.

## Introduction

### Background

Postacute COVID-19 syndrome, otherwise known as long COVID (LC), is a complex, multisystem disease that follows SARS-CoV-2 infection and often follows a relapsing and remitting course [1]. The postacute sequelae of LC could manifest with mild symptoms or asymptomatically. Although a distinct clinical phenotype remains to be defined, current evidence suggests that fatigue with postexertional symptom exacerbation is the most prominent, followed by shortness of breath, muscle aches, and cognitive impairment (brain fog) [2-4]. Risk factors are not well understood, and it appears that the characteristics that increase the risk of developing a severe COVID-19 infection (older age, male sex, non-White ethnicity, and certain pre-existing comorbidities) do not translate into an increased risk of developing LC [5]. Current research indicates that the prevalence of LC is greater amongst females, those aged 20-70 years, and those with prepandemic mental health conditions and asthma [6]. As the symptom pattern varies widely between individuals and risk factors have not been defined [7], it is difficult to establish an evidence-based framework for the recognition, assessment, and management of this condition.

In the United Kingdom, the Office for National Statistics (ONS) has estimated that 1.3 million people continue to have ongoing health issues after COVID-19 infection, with over 800,000 people reporting at least some limitation to their daily lives [2], although cases remain underrecorded in primary care electronic health records (EHRs) [8]. In December 2020 (updated in December 2021), the United Kingdom’s National Institute for Health and Care Excellence (NICE) recognized the lack of a clinical definition and released a rapid guideline [9]. NICE defines acute COVID-19 (symptoms lasting <4 weeks), ongoing symptomatic COVID-19 (symptoms lasting 4-12 weeks), and postacute COVID-19 syndrome (symptoms lasting >12 weeks), with the latter 2 considered as LC [3]. However, there remain limited treatment options or evidence-based rehabilitation guidance available for this condition, although research projects, such as the Long Covid Multidisciplinary Consortium: Optimising Treatments and Services across the National Health Service (NHS; LOCOMOTION), have been set up to address this [10].

Research on LC is confusing due to heterogenous study methods with minimal phenotypic information, and patient-reported symptoms often remain uncaptured [7]. Phenotypes are a standardized method for case definition and identification from routine data and are usually machine-processable. Computable phenotypes have become increasingly important in EHRs as they allow identification of patient characteristics using data that are generated during routine patient interactions [11]. An EHR-based phenotype definition is constructed by characterizing the disease in terms of its demographic profile, symptomatology, laboratory tests, and other clinically relevant data, such as referrals to specialist services [12]. This information can be displayed in the form of clinical codes or abstractly represented in the form of a logical data flow diagram [13]. In the United Kingdom, we use a national information standard, the Systematized Nomenclature of Medicine Clinical Terms (SNOMED-CT) and Read version 2 codes. It can then be written into a computational algorithm, which can be applied to EHRs to identify a specific cohort of patients. However, such a phenotype has to work within the constraints of data quality and clinical terminology used.

### Aims

The aim of this study is to develop a phenotype for LC using pseudonymized individual-level EHR data from English general practice that will enable the monitoring and evaluation of interventions for this condition. The specific objectives are:

- To develop a phenotype for LC
- To make this phenotype available in standard online formats in BioPortal and the PhenoFlow library
- To compare the symptoms reported by people with LC identified by the phenotype in the year prior to the pandemic with those they experienced during the pandemic
- To compare the symptoms of people with LC identified by the phenotype to those with acute COVID-19
- To compare people with LC identified by the phenotype who were hospitalized with those who were managed in the community

## Methods

### Data Source

The LC phenotype was piloted in an observational retrospective database analysis of the English Primary Care Sentinel Cohort (PCSC), which used data from the Oxford Royal College of General Practitioners (RCGP) Research and Surveillance Centre (RSC) sentinel network. This database is derived from pseudonymized patient data from EHRs and is recruited to be representative of the English population in terms of both demographic and clinical factors [14].

### Comparisons

**Figure 1:**
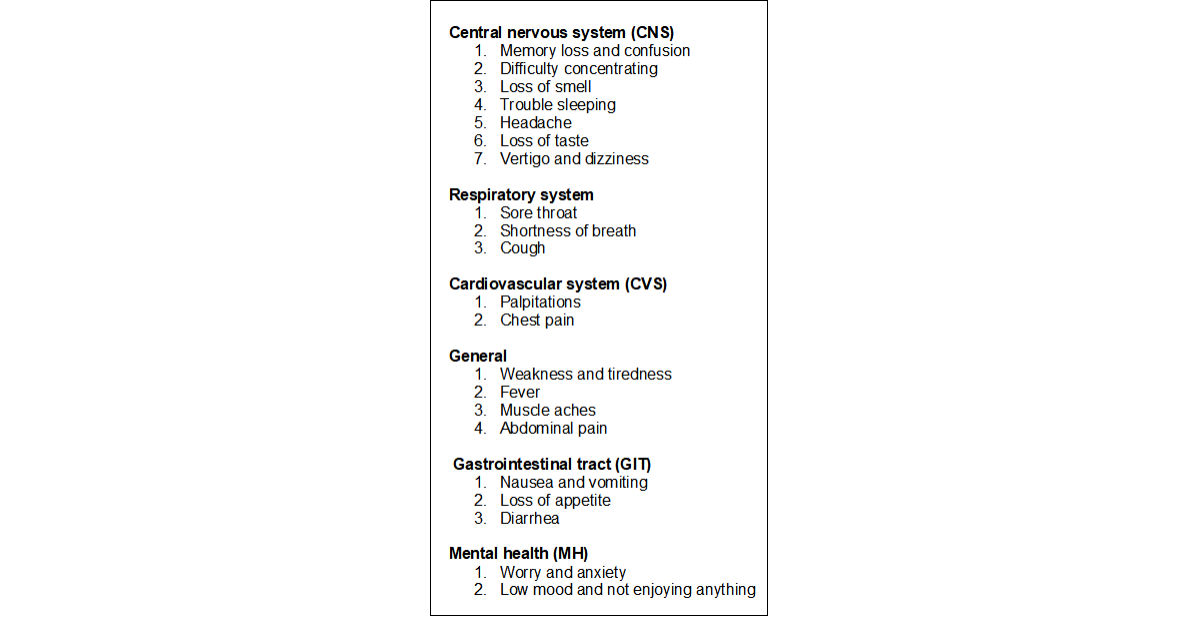
Symptoms identified by the UK Office for National Statistics (ONS) that are associated with long COVID (LC).

**Figure 2:**
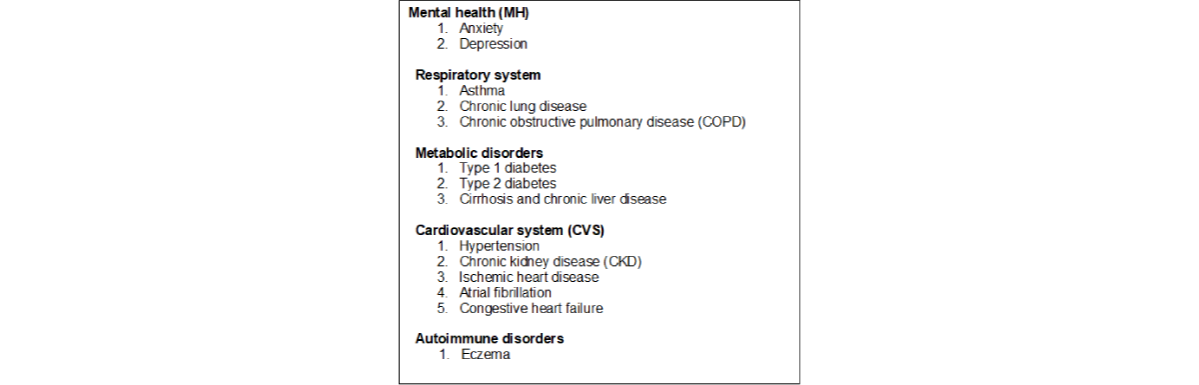
Summary of comorbidities included in our analyses.

This protocol piloted an LC phenotype in the PCSC and described the baseline characteristics and outcomes of those with LC. All people registered within the PCSC were eligible for inclusion in the study. The developed phenotype was used as a detailed reference for the inclusion and exclusion criteria. The study described further aspects of the epidemiology through 3 comparisons:

- Before-and-after symptom comparison in people with LC: We compared the presence of symptoms listed by the ONS between 1 and 6 months after index infection. We matched the period with the equivalent months for the previous year. The list of 21 symptoms developed by the ONS is broad and includes central nervous system symptoms, such as fatigue; respiratory symptoms; cardiovascular symptoms; general symptoms; gastrointestinal symptoms; and mental health symptoms (Figure 1). We defined an index date of COVID-19 hierarchically using our application ontology, which prioritized virologically proven cases (definite COVID-19) over clinical terms for a COVID-19–specific disease (probable COVID-19) over less definite clinical diagnoses (possible COVID-19) [15].
- Comparison of people with LC with those with acute COVID-19 uncomplicated by LC: We compared sociodemographic features, a range of comorbidities, vaccination status, and mortality between those who had LC and those who had a COVID-19 infection. Sociodemographic features included age; gender; ethnicity using 5 categories (Asian, Black, White, mixed, and others) [16]; socioeconomic status (SES), measured using the Index of Multiple Deprivation (IMD) [17]; population density divided into rural, town, city, and conurbation; the English Health Region; obesity, categorized by the BMI or the diagnostic clinical term into underweight, normal weight, overweight, obese, or severely obese; and, finally, smoking status, categorized into current smoker, ex-smoker, and nonsmoker. We conducted a literature review and identified a range of chronic diseases associated with the risk of COVID-19 complications (Figure 2) and an extended list differentiating long COVID and COVID-19 [1,5,8,18,19]. We reported the vaccination status stratified by the Cambridge Multimorbidity Score (CMMS) as an overall measure of multimorbidity [20]. The CMMS uses 37 conditions to predict primary care consultations, unplanned hospital admissions, and death as primary outcomes; it is useful to identify people who are at higher risk of specific outcomes based on their comorbidity profiles, as recorded in primary care EHR data.
- Comparison of those with LC who were hospitalized with those who were not: We used the same variables to compare people who were hospitalized and subsequently had LC with those who were not hospitalized but had LC diagnosed in the community. We conducted a sensitivity analysis where we subdivided the community cases into 2 groups: people who had an index COVID-19 infection either virologically confirmed or sometimes clinically diagnosed and those who have an LC diagnosis, a referral to an LC service, or a LC disability rating score compatible with an LC diagnosis (eg, Yorkshire LC score) [21].

### Phenotype Development

We used a 3-step ontological approach to create our phenotype [12], considering ontological, coding, and logical layers.

#### Ontological Layer

The key concept identified in our ontological layer was an index date for COVID-19, noting that not all cases had virological confirmation (especially in the early part of the pandemic up to July 2020). Hence, some LC cases might only have been flagged on referral or later presentation. We wanted to also include whether cases were hospitalized, as hospitalization can be associated with poor outcomes [22]. Additionally, we included vaccination status to explore if protective.

#### Coding Layer

We applied our existing ontology to identify COVID-19 cases. We included key outcomes related to hospital admissions. These were any hospitalization, admission to intensive care, or death in the hospital. To be a case of LC, we included disease codes, primarily recorded with SNOMED-CT or the World Health Organization’s (WHO) *International Classification of Diseases* (ICD). The clinical term could be a diagnosis, a referral (eg, referral to post–COVID-19 assessment clinic), or completion of a rating scale that implied LC (eg, the Yorkshire Rehabilitation Scale, which records symptom severity, functional disability, and health status) [23].

#### Logical Data Extraction Model

We planned our data extraction using pseudonymized primary care data. We supplemented these data with national data sets. The national data sets used were the Second Generation Surveillance System (SGSS) to capture any missing test data, the National Immunisation Management System (NIMS) to capture any missing vaccine recording, and Hospital Episode Statistics (HES) to add hospital outcome data. The ONS also provided death data. We pseudonymized all data as close to the source as possible using an NHS Digital–approved method. We used the same pseudonymization method to link primary care data to other data sources.

**Figure 3:**
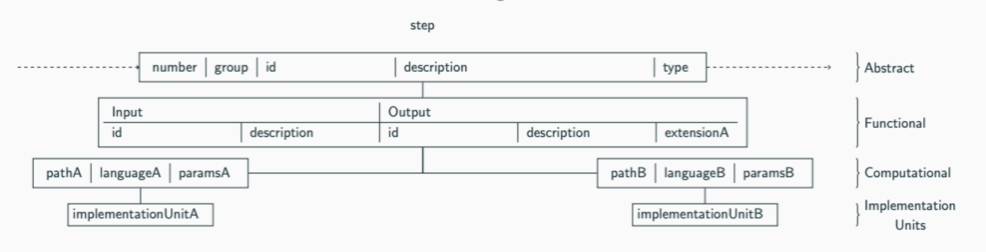
PhenoFlow multilayer model describes each step within the multistep phenotype definition contained within the phenotype.

**Figure 4:**
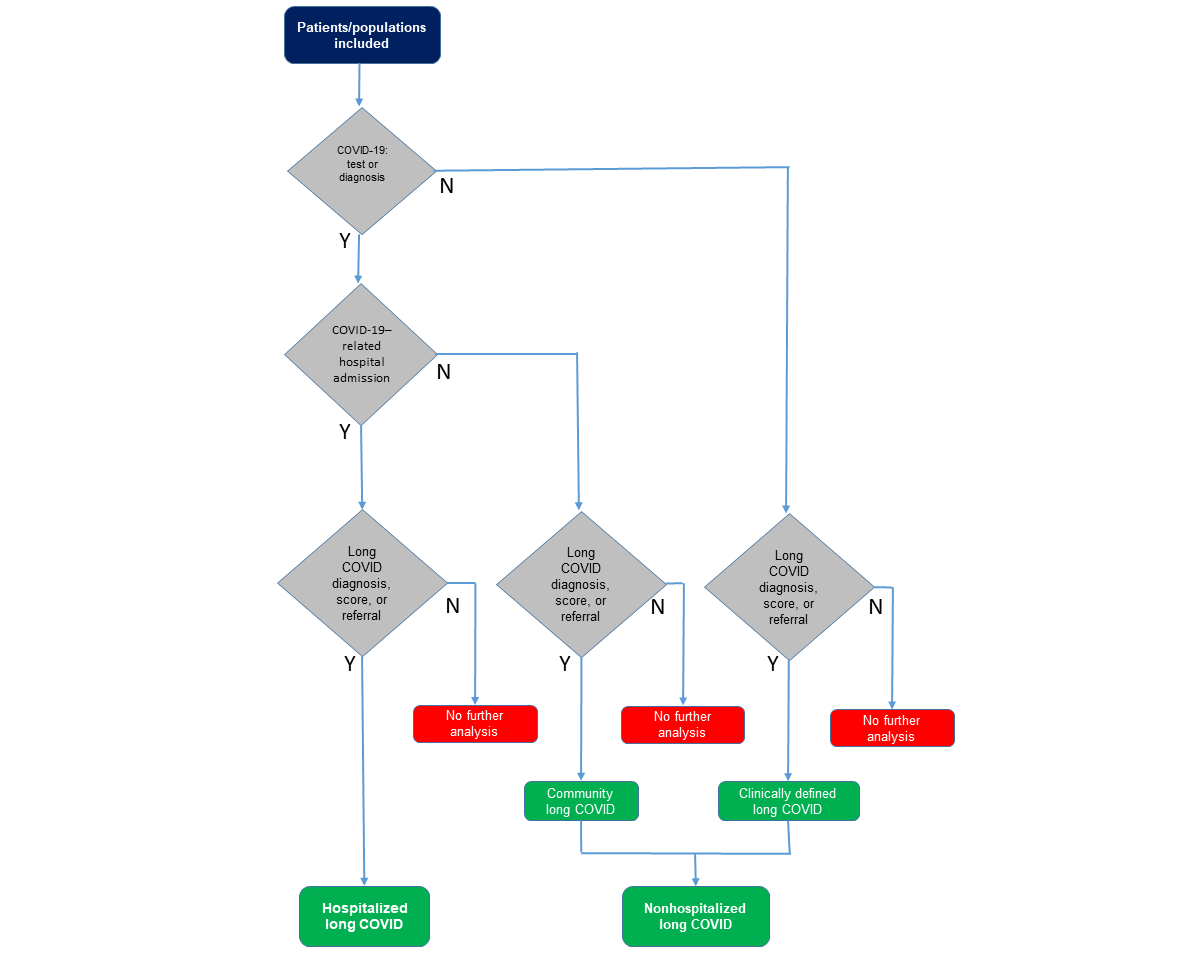
Logic model for the LC phenotype. LC: long COVID.

Our phenotype definition is presented as a structured multistep model (Figure 3) and as a logic model (Figure 4). This omitted the reporting of vaccine exposure by group.

#### Formal Ontologies: BioPortal and PhenoFlow

From the logic model, we created 2 formal ontologies. We used Protégé, an open source ontology editor, to construct a domain ontology, which we placed online via BioPortal. Protégé supports Ontology Web Language (OWL) version 2 and Resource Description Frameworks (RDFs) from the World Wide Web Consortium (W3C) [24]. BioPortal is part of the National Center for Biomedical Ontology (in the United States) that supports the creation of interoperable ontologies.

We also created a version within the PhenoFlow library [13]. The PhenoFlow library imports and standardizes abstract definitions under a workflow-based multilayer model, which is later used as the basis for autonomously generating a computable form of the definition. This can then be downloaded and executed locally to identify a patient cohort. Standardizing a definition under the PhenoFlow model also assists with manual phenotype translation as it supplements the use of clinical terminology and simplifies the representation of logical structures, thus increasing intelligibility (Figure 3). The model allows greater flexibility in updating phenotypes and also increases portability.

The model consisted of 3 layers and included the type or classification of the step’s logic, with detailed information regarding inputs and outputs at each relevant step. This information was combined with 1 or more implemented units (eg, a piece of Python code) in order to realize a computable phenotype.

### Statistical Analysis

This study is a secondary analysis of existing pseudonymized data within the PCSC of the RSC. Although we noted that 58 (7.8%) of 743 practices had not recorded any LC cases in their EHR system, they were included in the analysis as it is likely that recording would improve during the course of the study, with increased interest in this condition [25].

The distribution of baseline characteristics among the study groups was summarized through descriptive statistics (eg, mean, median, and proportion) with measures of dispersion (eg, SD and IQRs). Univariate analyses included the calculation of odds ratios (ORs) for categorical risk factors versus outcome levels with 95% CIs by using the log(p/1 – p) link function. Logarithmic transformation of the outcome variable allowed a nonlinear association in a linear manner. *P* values were obtained from a chi-square test for categorical variables and one-way ANOVA for continuous variables. Data that were not documented in our database were reported as missing.

The primary outcome measure was the association with LC using our phenotype. Multivariate logistic regression modeling was used to identify factors associated with LC as a binary outcome within the study population. Relevant risk factors identified in the literature underwent univariate analysis and were included in multivariate logistic regression using a 3-step backward elimination procedure with of α threshold levels of 0.20, 0.10, and 0.05. A 2-sided α value of 0.05 was considered statistically significant. Missing data were presented as a separate category in univariate statistics and compared to the reference category. Missing data categories were imputed to the reference category if no significant differences were found in the reference category. Missing data categories were otherwise included in multivariate regression as a separate category, under the assumption that they may not be missing at random.

The following 3 comparisons were made, reporting frequencies between groups with *P* values obtained from the chi-square test:

- Symptoms reported by people with LC in the year prior to the pandemic versus those they experienced during the pandemic. The study period included COVID-19 cases from March 1, 2020, to April 1, 2021, with a follow-up period of a further 6 months up to, latest, September 30, 2021. This historical comparator period was month-matched; for example, if a patient had an acute COVID-19 code entered on February 1, 2021, their follow-up period was March 1-July 31, 2021, and the historic comparator period was March 1-July 31, 2019. This allowed the comparison of rates of relevant symptoms prior to having acute COVID-19 with after having acute COVID-19. The in-pandemic period was between 1 and 6 months after their index COVID-19 date. For those without a COVID-19 index date, we compared the 5 months prior to their LC recording with a matched period in the previous year.
- Symptoms of people with LC versus those with acute COVID-19. Although we accepted that LC is underrecorded, we considered this analysis of importance as the phenotype of those recorded was likely to be similar to those unrecorded, although potentially with more prominent or debilitating symptomatology.
- Those hospitalized with LC versus those managed in the community. A final comparison was then made between people requiring hospital admission for acute COVID-19 and those who were managed in the community and people who had no documented evidence of acute COVID-19. We also included a comparison between community cases with and without an index infection.

These comparisons enabled us to explore how the clinical phenotype varies. We also reported the vaccination uptake between people with and without LC diagnosis.

### Ethical Considerations

This study used existing data, and no subjects were recruited. RSC data used to create this phenotype were pseudonymized as close to the source as possible and sent in an encrypted format to the Oxford Royal College of General Practitioners Clinical Informatics Digital Hub (ORCHID) [15], which is recognized as a trusted research environment.

This study was part of the RECAP (Predicting Risk of Hospital Admission in Patients with Suspected COVID-19 in a Community Setting) study sponsored by the Imperial College London [26]. Although primarily a study to develop a risk prediction tool, it also included the creation of an LC phenotype. Ethical approval was granted by the North West–Greater Manchester East Research Ethics Committee and Health Research Authority on May 27, 2021 (Integrated Research Application System #283024, Research Ethics Committee reference #20/NW/0266).

## Results

### Phenotype: Logic Model

The logic model for the phenotype is shown in Figure 4. It depicts the hierarchical structure for identifying LC cases from the ontological layer of EHR data. The ontology logic runs hierarchically, first screening the population for COVID-19 cases (ie, firm diagnosis of acute COVID-19). Those with an index COVID-19 case were then screened for COVID-19–related hospital admissions. When no index COVID-19 cases were documented, the model still allowed for LC cases to be included as long as they had an entry within their EHRs, implying they had LC (ie, clinically defined LC).

### Phenotype: BioPortal and PhenoFlow

**Figure 5:**
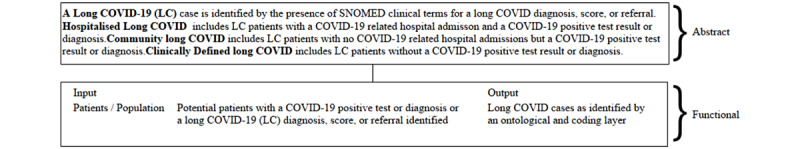
Individual steps of the LC phenotype definition logic. LC: long COVID.

The LC phenotype definition was built in Protégé, which is an open source ontology editor that supports the latest OWL. This phenotype was then uploaded to BioPortal. The LC phenotype definition (Figure 5) can be accessed online [27] and provides a framework for researchers wanting to develop their own executable script to apply to databases.

Within BioPortal, the ontological layer of the structured phenotype is described within a class and subclass structure, while the coding layer is represented by individuals within each class and subclass. BioPortal ontologies can be readily updated.

**Figure 6:**
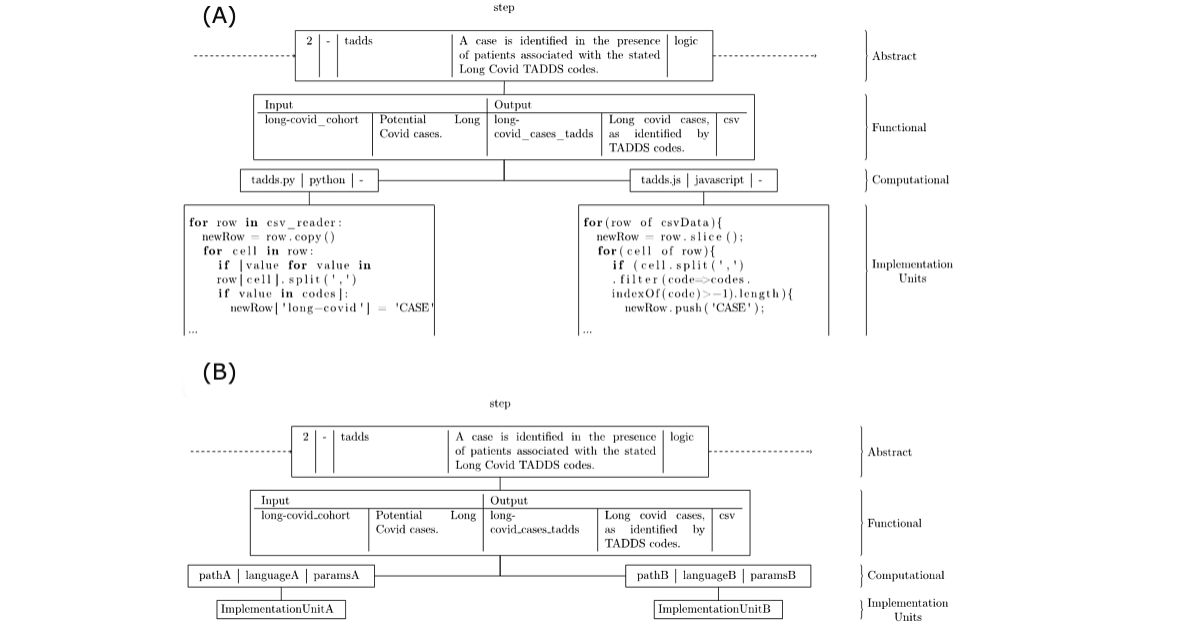
LC PhenoFlow model. (A) Individual steps of LC structured phenotype definition with and without implementation units and (B) individual steps of a structured phenotype using ORCHID-themed variable (TADDS) codes and implementation units. LC: long COVID; ORCHID: Oxford Royal College of General Practitioners Clinical Informatics Digital Hub.

The PhenoFlow library was used to transform the LC phenotype into a computable form (Figure 6). The LC phenotype can be accessed online with authorization [28], it can be downloaded, and, unlike BioPortal, it is ready for researchers to apply to EHR databases.

### Primary Care Sentinel Cohort

The PCSC of the RSC has a registered population of over 7 million (N=7,382,775). At the time of our data extraction, 428,479 (5.8%) of this population had an acute episode of COVID-19 recorded. Of this group, 42,321 (9.9%) were lost to follow-up; 40% (n=16,993) of this loss to follow-up was due to deaths, with just under half of these deaths (7531/16,993, 44.3%) being COVID-19 related. A total of 403,151 (94.1%) cases were included in the analysis, of whom 19,002 (4.7%) were hospitalized and 384,149 (95.3%) were not.

### People With LC

**Figure 7:**
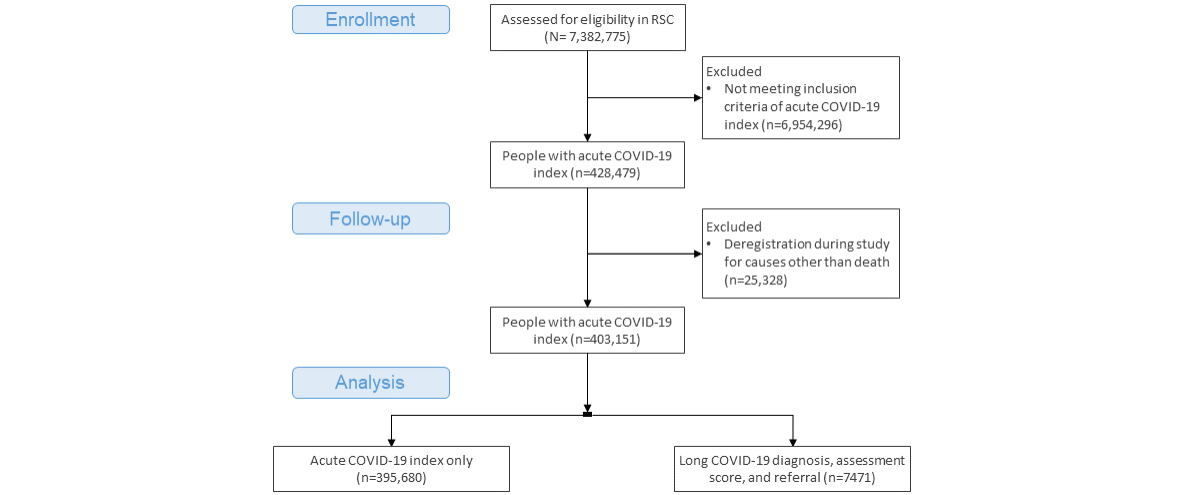
Consolidated Standards of Reporting Trials (CONSORT) diagram for the study population.

We identified 7471 (1.8%) of 428,479 people recorded as having LC within this included group (Figure 7). A greater proportion were hospitalized in the LC group compared to the overall hospitalization rate (1009/7471, 13.5%, *P*<.001). Within this group, there were a small number of deaths (23/7471, 0.3%, *P*<.001).

### Comparison of People With Acute COVID-19 and LC

We paired data for people with COVID-19 (n=395,680, 98.1%) and LC (n=7471, 1.9%), expecting to perform comparisons of baseline characteristics between both groups. Among the main preliminary findings, the mean age was 44.6 (SD 21.75) years for the COVID-19 group and 47.7 (SD 14.8) years for the LC group. A significantly higher proportion of those with LC were found among females (4836/7471, 64.7%), where the male gender was associated with lower odds for LC (OR 0.68, 95% CI 0.65-0.72). The proportion of those with a record of intensive care unit (ICU) admission was 0.6% (2523/395,680) in people with COVID-19 and 3.5% (261/7471) in people with LC, where a record of ICU admission was associated with higher odds of LC (OR 5.64, 95% CI 4.96-6.42). Sociodemographic characteristics reporting higher odds for LC in the univariate analysis included living in a conurbation (OR 1.49, 95% CI 1.42-1.57) and obesity (OR 1.40, 95% CI 1.34-1.48). Comorbidities associated with higher odds of LC included depression, anxiety, asthma, and hypertension. In contrast, chronic lung disease, chronic obstructive pulmonary disease (COPD), chronic kidney disease (CKD), ischemic heart disease, atrial fibrillation, and congestive heart failure were associated with lower odds of LC.

The baseline characteristics of the population are shown in Tables 1-4.

**Table 1:** Frequencies of baseline characteristics and univariate ORs^a^ (sociodemographics) for people with COVID-19 stratified by long COVID (LC) status in the PCSC^b^ in England (March 1, 2020-April 1, 2021).

Variable and category			Overall (N=403,151)		COVID-19 (n=395,680)		LC (n=7471)		OR (95% CI)		*P* value
Age (years; continuous), mean (SD)			44.62 (21.65)		44.56 (21.75)		47.74 (14.81)		1.01 (1.01-1.01)		<.001
**Gender, n (%)**											
	Female (Ref.^c^)	224,934 (55.8)		220,098 (55.6)		4836 (64.7)		1.00 (N/A^d^)		N/A	
	Male	178,217 (44.2)		175,582 (44.4)		2635 (35.3)		0.68 (0.65-0.72)		<.001	
**Deprivation, n (%)**											
	Least deprived (Ref.)	156,115 (38.7)		153,198 (38.7)		2917 (39.0)		1.00 (N/A)		N/A	
	Most deprived	229,986 (57.0)		225,608 (57.0)		4378 (58.6)		1.02 (0.97-1.07)		.43	
	Missing	17,050 (4.2)		16,874 (4.3)		176 (2.4)		0.55 (0.47-0.64)		<.001	
**Ethnicity, n (%)**											
	White (Ref.)	262,878 (65.2)		257,507 (65.1)		5371 (71.9)		1.00 (N/A)		N/A	
	Non-White	55,609 (13.8)		54,549 (13.8)		1060 (14.2)		0.93 (0.87-1.00)		.04	
	Missing	84,664 (21.0)		83,624 (21.1)		1040 (13.9)		0.60 (0.56-0.64)		<.001	
**Ethnicity point, n (%)**											
	Asian	35,214 (8.7)		34,587 (8.7)		627 (8.4)		N/A		<.001	
	Black	11,286 (2.8)		11,022 (2.8)		264 (3.5)		N/A		N/A	
	Other	9109 (2.3)		8940 (2.3)		169 (2.3)		N/A		N/A	
	Unknown	84,664 (21.0)		83,624 (21.1)		1040 (13.9)		N/A		N/A	
	White	262,878 (65.2)		257,507 (65.1)		5371 (71.9)		N/A		N/A	
**Population density, n (%)**											
	City (Ref.)	201,839 (50.1)		198,709 (50.2)		3130 (41.9)		1.00 (N/A)		N/A	
	Conurbation	136,068 (33.8)		132,942 (33.6)		3126 (41.8)		1.49 (1.42-1.57)		<.001	
	Rural	65,244 (16.2)		64,029 (16.2)		1215 (16.3)		1.20 (1.13-1.29)		<.001	
**NHS^e^ region, n (%)**											
	London	55,191 (13.7)		53,721 (13.6)		1470 (19.7)		N/A		<.001	
	Midlands	67,375 (16.7)		66,405 (16.8)		970 (13.0)		N/A		N/A	
	North and east	95,209 (23.6)		93,995 (23.8)		1214 (16.2)		N/A		N/A	
	Northwest	67,211 (16.7)		65,768 (16.6)		1443 (19.3)		N/A		N/A	
	South	118,165 (29.3)		115,791 (29.3)		2374 (31.8)		N/A		N/A	
**BMI, n (%)**											
	Nonobese (Ref.)	247,957 (61.5)		243,520 (61.5)		4437 (59.4)		1.00 (N/A)		N/A	
	Obese	101,194 (25.1)		98,669 (24.9)		2525 (33.8)		1.40 (1.34-1.48)		<.001	
	Missing	54,000 (13.4)		53,491 (13.5)		509 (6.8)		0.52 (0.48-0.57)		<.001	
**Smoker, n (%)**											
	Nonsmoker (Ref.)	207,788 (51.5)		203,422 (51.4)		4366 (58.4)		1.00 (N/A)		N/A	
	Smoker/ex-smoker	147,819 (36.7)		144,930 (36.6)		2889 (38.7)		0.93 (0.89-0.97)		<.001	
	Missing	47,544 (11.8)		47,328 (12.0)		216 (2.9)		0.21 (0.19-0.24)		<.001	

^a^OR: odds ratio.

^b^PCSC: Primary Care Sentinel Cohort.

^c^Ref.: reference category.

^d^N/A: not applicable.

^e^NHS: National Health Service.

**Table 2:** Frequencies of baseline characteristics and univariate ORs^a^ (comorbidities) for people with COVID-19 stratified by LC^b^ status in the PCSC^c^ in England (March 1, 2020-April 1, 2021).

Variable and category			Overall (N=403,151)		COVID-19 (n=395,680)		LC (n=7471)		OR (95% CI)		*P* value
**Depression, n (%)**											
	No (Ref.^d^)	310,251 (77.0)		305,486 (77.2)		4765 (63.8)		1.00 (N/A^e^)		N/A	
	Yes	92,900 (23.0)		90,194 (22.8)		2706 (36.2)		1.92 (1.83-2.02)		<.001	
**Anxiety, n (%)**											
	No (Ref.)	308,752 (76.6)		303,878 (76.8)		4874 (65.2)		1.00 (N/A)		N/A	
	Yes	94,399 (23.4)		91,802 (23.2)		2597 (34.8)		1.76 (1.68-1.85)		<.001	
**Asthma, n (%)**											
	No (Ref.)	328,133 (81.4)		322,434 (81.5)		5699 (76.3)		1.00 (N/A)		N/A	
	Yes	75,018 (18.6)		73,246 (18.5)		1772 (23.7)		1.37 (1.30-1.44)		<.001	
**Chronic lung disease, n (%)**											
	No (Ref.)	388,932 (96.5)		381,651 (96.5)		7281 (97.5)		1.00 (N/A)		N/A	
	Yes	14,219 (3.5)		14,029 (3.5)		190 (2.5)		0.71 (0.61-0.82)		<.001	
**COPD^f^, n (%)**											
	No (Ref.)	390,544 (96.9)		383,219 (96.9)		7325 (98.0)		1.00 (N/A)		N/A	
	Yes	12,607 (3.1)		12,461 (3.1)		146 (2.0)		0.61 (0.52-0.72)		<.001	
**Hypertension, n (%)**											
	No (Ref.)	322,978 (80.1)		317,094 (80.1)		5884 (78.8)		1.00 (N/A)		N/A	
	Yes	80,173 (19.9)		78,586 (19.9)		1587 (21.2)		1.09 (1.03-1.15)		<.003	
**CKD^g^, n (%)**											
	No (Ref.)	380,699 (94.4)		373,496 (94.4)		7203 (96.4)		1.00 (N/A)		N/A	
	Yes	22,452 (5.6)		22,184 (5.6)		268 (3.6)		0.63 (0.55-0.71)		<.001	
**Ischemic heart disease, n (%)**											
	No (Ref.)	381,585 (94.7)		374,450 (94.6)		7135 (95.5)		1.00 (N/A)		N/A	
	Yes	21,566 (5.3)		21,230 (5.4)		336 (4.5)		0.83 (0.74-0.93)		<.001	
**Atrial fibrillation, n (%)**											
	No (Ref.)	389,749 (96.7)		382,408 (96.6)		7341 (98.3)		1.00 (N/A)		N/A	
	Yes	13,402 (3.3)		13,272 (3.4)		130 (1.7)		0.51 (0.43-0.61)		<.001	
**Congestive heart failure, n (%)**											
	No (Ref.)	395,034 (98.0)		387,625 (98.0)		7409 (99.2)		1.00 (N/A)		N/A	
	Yes	8117 (2.0)		8055 (2.0)		62 (0.8)		0.40 (0.31-0.52)		<.001	
**Type 2 diabetes, n (%)**											
	No (Ref.)	372,818 (92.5)		365,912 (92.5)		6906 (92.4)		1.00 (N/A)		N/A	
	Yes	30,333 (7.5)		29,768 (7.5)		565 (7.6)		1.01 (0.92-1.10)		.90	
	Yes	13,402 (3.3)		13,272 (3.4)		130 (1.7)		0.51 (0.43-0.61)		<.001	
**Type 1 diabetes, n (%)**											
	No (Ref.)	400,625 (99.4)		393,196 (99.4)		7429 (99.4)		1.00 (N/A)		N/A	
	Yes	2526 (0.6)		2484 (0.6)		42 (0.6)		0.89 (0.66-1.22)		.47	
**Cirrhosis, n (%)**											
	No (Ref.)	402,118 (99.7)		394,663 (99.7)		7455 (99.8)		1.00 (N/A)		N/A	
	Yes	1033 (0.3)		1017 (0.3)		16 (0.2)		0.83 (0.51-1.37)		.46	
**Eczema, n (%)**											
	No (Ref.)	313,703 (77.8)		307,931 (77.8)		5772 (77.3)		1.00 (N/A)		N/A	
	Yes	89,448 (22.2)		87,749 (22.2)		1699 (22.7)		1.03 (0.98-1.09)		.25	

^a^OR: odds ratio.

^b^LC: long COVID.

^c^PCSC: Primary Care Sentinel Cohort.

^d^Ref.: reference category.

^e^N/A: not applicable.

^f^COPD: chronic obstructive pulmonary disease.

^g^CKD: chronic kidney disease.

**Table 3:** Frequencies of baseline characteristics and univariate ORs^a^ (exposures) for people with COVID-19 stratified by LC^b^ status in the PCSC^c^ in England (March 1, 2020-April 1, 2021).

Variable and category			Overall (N=403,151)		COVID-19 (n=395,680)		LC (n=7471)		OR (95% CI)		*P* value
**Hospitalized, n (%)**											
	No (Ref.^d^)	384,149 (95.3)		377,687 (95.5)		6462 (86.5)		1.00 (N/A^e^)		N/A	
	Yes	19,002 (4.7)		17,993 (4.5)		1009 (13.5)		3.28 (2.95-3.38)		<.001	
**ICU^f^ admission, n (%)**											
	No (Ref.)	400,367 (99.3)		393,157 (99.4)		7210 (96.5)		1.00 (N/A)		N/A	
	Yes	2784 (0.7)		2523 (0.6)		261 (3.5)		5.64 (4.96-6.42)		<.001	
**COVID-19 vaccination at any point, n (%)**											
	No vaccine (Ref.)	81,081 (20.1)		80,229 (20.3)		852 (11.4)		1.00 (N/A)		N/A	
	One dose	25,018 (6.2)		24,655 (6.2)		363 (4.9)		1.39 (1.23-1.57)		<.001	
	Two doses	297,052 (73.7)		290,796 (73.5)		6256 (83.7)		2.03 (1.89-2.18)		<.001	
**First vaccination brand, n (%)**											
	AstraZeneca	168,444 (41.8)		164,652 (41.6)		3792 (50.8)		N/A		<.001	
	Pfizer-BioNTech	146,328 (36.3)		143,647 (36.3)		2681 (35.9)		N/A		N/A	
	Other	6986 (1.7)		6843 (1.7)		143 (1.9)		N/A		N/A	
	None	81,393 (20.2)		80,538 (20.4)		855 (11.4)		N/A		N/A	
**Second vaccination brand, n (%)**											
	AstraZeneca	163,206 (40.5)		159,541 (40.3)		3,665 (49.1)		N/A		<.001	
	Pfizer-BioNTech	127,048 (31.5)		124,604 (31.5)		2444 (32.7)		N/A		N/A	
	Other	6279 (1.6)		6150 (1.6)		129 (1.7)		N/A		N/A	
	None	106,605 (26.4)		105,372 (26.6)		1233 (16.5)		N/A		N/A	
	N/A	13 (0)		13 (0)		0		N/A		N/A	

^a^OR: odds ratio.

^b^LC: long COVID.

^c^PCSC: Primary Care Sentinel Cohort.

^d^Ref.: reference category.

^e^N/A: not applicable.

^f^ICU: intensive care unit.

**Table 4:** Frequencies of baseline characteristics and univariate ORs^a^ (mortality) for people with COVID-19 stratified by LC^b^ status in the PCSC^c^ in England (March 1, 2020-April 1, 2021).

Variable and category			Overall (N=403,151)		COVID-19 (n=395,680)		LC (n=7471)		OR (95% CI)		*P* value
**All-cause mortality, n (%)**											
	No (Ref.^d^)	386,158 (95.8)		378,710 (95.7)		7448 (99.7)		1.00 (N/A^e^)		N/A	
	Yes	16,993 (4.2)		16,970 (4.3)		23 (0.3)		0.07 (0.05-0.10)		<.001	

^a^OR: odds ratio.

^b^LC: long COVID.

^c^PCSC: Primary Care Sentinel Cohort.

^d^Ref.: reference category.

^e^N/A: not applicable.

### Comparison of Those Hospitalized and Those Not Hospitalized

For the group of people with LC, we paired data for people with a record of hospitalization (n=1009, 13.5%) and without hospitalization (n=6462, 86.5%). The mean age was 54.6 (SD 13.69) years for the hospitalized group and 46.7 (SD 14.7) years for the nonhospitalized group, while the proportion of females was 66.5% (4297/6462) in the nonhospitalized group and 53.4% (539/1009) in the hospitalized group. Factors associated with greater odds of hospitalization were the male gender (OR 1.73, 95% CI 1.51-1.98) and type 2 diabetes (OR 3.8, 95% CI 3.15-4.59).

## Discussion

### Principal Findings

We created a phenotype for LC and made it publicly available with the aim of facilitating research in this area. Our phenotype is straightforward but based on the presence of a postacute COVID-19 syndrome code being present in the EHR. The definition allows comparison of hospitalized and nonhospitalized groups and the inclusion of people with no baseline COVID-19 test data. Our phenotype’s logical model can also allow vaccine exposure to be compared between groups.

Based on our network data, LC recording within primary care appears to be low and we noted interpractice variability, with some practices (8%) having no recorded cases. It was not possible to generate a symptom-related definition that might help close the gap between the level of recording in primary care and that identified through the ONS surveys [2].

Many different conditions have been associated with LC, and we made pragmatic, literature-based choices regarding which groups we should contrast where we make LC comparisons. We consider that before-and-after, acute COVID-19 compared with LC and hospitalized compared with nonhospitalized LC analyses will provide an assessment of our phenotype’s performance and face validity.

Digitization of health systems worldwide has led to the emergence of EHR repositories for the study of both established and emerging diseases and trends. Phenotyping algorithms allow identification of patients within EHRs who share characteristics, and therefore play an important role in medical cohort studies. High-quality phenotypes must be portable, accessible, and reproducible. A number of phenotype libraries have been developed or are undergoing development [29] in order to collect and store validated phenotype definitions. Our LC phenotype is available to download from BioPortal, where researchers can use it to produce their own executable script. By additionally applying the phenotype using the PhenoFlow model with “functional” and “computational” layers, our phenotype goes 1 step further with the capability for immediate execution in EHRs. As the characteristics of LC change with more data, vaccines, and treatments becoming available, the flexibility of the PhenoFlow model allows the phenotype to be readily updated and reapplied.

### Comparison With Prior Work

Applying the phenotype within the RSC, we identified 7471 patients with LC. The LC group was older overall, more likely to be female, obese, and suffering from anxiety, depression, or asthma. These findings are in keeping with studies using patient-reported data and EHRs [5,6]. In the acute COVID-19 group, 17,993 (4.5%) of 395,680 patients were hospitalized. The number of patients hospitalized with COVID-19 in the LC group was much higher (1009/7471, 13.5%). Furthermore, patients with LC were more likely to have had an ICU admission: 261/7471 (3.5%) versus 2523/395,680 (0.6%). Similar findings were reported by O’Connor et al [23] in an observational study of 187 patients with 15% hospitalized and 5.4% admitted to the ICU. The Zoe Symptom Study app [5] reports even higher rates of patients attending the hospital (up to 44% of those experiencing symptoms for more than 56 days) but does not clarify whether these patients were admitted to the hospital. Survey studies such as these may also suffer from selection bias and are not necessarily representative of the wider population. Nevertheless, hospital attendance during the acute infection appears to be a risk factor for LC, and further work is required to address this.

### Strengths and Limitations

Our study used the PCSC of the RSC, 1 of Europe’s oldest sentinel systems and one widely involved in pandemic research [14,15,30]. Data quality is good, and linkage to national registries ensured reliable data, including mortality [31]. Additionally, UK primary care is universal and a registration-based system. Nearly all emergency care is provided by the NHS, and national systems enable capture of COVID-19 tests and vaccination data.

The complexity of LC and its multiple symptoms and associations made this analysis challenging. We were selective based on the literature available on the conditions we compared. The statistical analysis was limited to establishing associations between known covariates and outcomes, testing the face validity our LC phenotype against other reports in the United Kingdom. Further research should explore causality of the reported findings under appropriate study designs.

We likely underestimated the frequency of ongoing symptoms following acute infection, because many people do not seek medical care for these. There were also, like all studies using routine data, some issues with data quality. For example, clinicians may have “coded” (used clinical terms) based on symptoms (eg, fatigue) rather than using a “long COVID-19” clinical term to “code” this illness. It is also possible that key data were not coded at all but were included in the free-text narrative within EHRs. Our study aimed to compare LC in the hospitalized and nonhospitalized groups. It is possible that these represent 2 separate populations with different symptom clusters. Those hospitalized with acute COVID-19 are more likely to suffer from respiratory and other organ damage, whereas those managed in the community may suffer from a potentially different range of LC symptoms with a lower risk of end-organ damage and mortality. The lack of fine-detailed symptom categorization in EHRs may have limited this comparison. Symptom coding was also impacted by clinicians’ cognitive biases, a known limitation of epidemiological research using routinely recorded data [32].

Finally, LC clinical terms were only added to SNOMED in January 2021 and thus would not have become available in EHRs until around February 2021, almost a year after the onset of the pandemic. The United Kingdom also has its own version of SNOMED-CT, and there are a range of different clinical terms available internationally.

Further research is required to explore symptom clusters and assess key differences in those hospitalized compared to those managed in the community.

### Conclusion

Developing and validating an LC phenotype will enable the identification of individuals with the condition and facilitate comparison between affected and unaffected people. However, LC is a complex condition with a wide variety of symptoms that will require further research to understand. This phenotype and study protocol to explore its face validity should contribute to a better understanding of LC.

## Abbreviations

CMMS: Cambridge Multimorbidity Score
EHR: electronic health record
ICU: intensive care unit
LC: long COVID
NHS: National Health Service
NICE: National Institute for Health and Care Excellence
ONS: Office for National Statistics
OR: odds ratio
ORCHID: Oxford Royal College of General Practitioners Clinical Informatics Digital Hub
OWL: Ontology Web Language
PCSC: Primary Care Sentinel Cohort
RSC: Research and Surveillance Centre
RCGP: Royal College of General Practitioners
SNOMED-CT: Systematized Nomenclature of Medicine Clinical Terms
